# The GTPase RalA Regulates Different Steps of the Secretory Process in Pancreatic β-Cells

**DOI:** 10.1371/journal.pone.0007770

**Published:** 2009-11-05

**Authors:** Sanda Ljubicic, Paola Bezzi, Nicolas Vitale, Romano Regazzi

**Affiliations:** 1 Department of Cell Biology and Morphology, University of Lausanne, Lausanne, Switzerland; 2 Département Neurotransmission et Sécrétion Neuroendocrine, Institut des Neurosciences Cellulaires et Intégratives (UPR-3212) CNRS and University of Strasbourg, Strasbourg, France; University of Bremen, Germany

## Abstract

**Background:**

RalA and RalB are multifuntional GTPases involved in a variety of cellular processes including proliferation, oncogenic transformation and membrane trafficking. Here we investigated the mechanisms leading to activation of Ral proteins in pancreatic β-cells and analyzed the impact on different steps of the insulin-secretory process.

**Methodology/Principal Findings:**

We found that RalA is the predominant isoform expressed in pancreatic islets and insulin-secreting cell lines. Silencing of this GTPase in INS-1E cells by RNA interference led to a decrease in secretagogue-induced insulin release. Real-time measurements by fluorescence resonance energy transfer revealed that RalA activation in response to secretagogues occurs within 3–5 min and reaches a plateau after 10–15 min. The activation of the GTPase is triggered by increases in intracellular Ca^2+^ and cAMP and is prevented by the L-type voltage-gated Ca^2+^ channel blocker Nifedipine and by the protein kinase A inhibitor H89. Defective insulin release in cells lacking RalA is associated with a decrease in the secretory granules docked at the plasma membrane detected by Total Internal Reflection Fluorescence microscopy and with a strong impairment in Phospholipase D1 activation in response to secretagogues. RalA was found to be activated by RalGDS and to be severely hampered upon silencing of this GDP/GTP exchange factor. Accordingly, INS-1E cells lacking RalGDS displayed a reduction in hormone secretion induced by secretagogues and in the number of insulin-containing granules docked at the plasma membrane.

**Conclusions/Significance:**

Taken together, our data indicate that RalA activation elicited by the exchange factor RalGDS in response to a rise in intracellular Ca^2+^ and cAMP controls hormone release from pancreatic β-cell by coordinating the execution of different events in the secretory pathway.

## Introduction

Insulin secretion from pancreatic β-cells is essential to maintain tight control of blood glucose levels [1]. Defects in this process can lead to chronic hyperglycaemia and to the development of diabetes mellitus. In β-cells, the increase in intracellular ATP/ADP ratio resulting from glucose metabolism causes closure of ATP-sensitive K^+^-channels and membrane depolarization [1]. This triggers opening of voltage-gated Ca^2+^ channels and elevation of intracellular Ca^2+^ concentrations ([Ca^2+^]_i_). The increase in [Ca^2+^]_i_ is both necessary and sufficient to elicit an initial burst of insulin exocytosis, mediated by fusion of insulin granules docked at the plasma membrane. [Ca^2+^]_i_ elevation is also necessary for a second, long-lasting phase of insulin exocytosis involving mobilization of secretory granules from a reserve pool. In this case, secretion is sustained by mitochondrial signals generated from glucose metabolism. Glucose is the main stimulus for insulin release but the secretory process can be finely tuned by second messengers such as cAMP and diacylglycerol that are generated in response to changes in the concentrations of nutrients, hormones and neurotransmitters. Despite recent progress in the identification of the components of the molecular machinery driving insulin exocytosis, the precise mechanisms through which second messenger generation is coupled to the activation of the secretory process are still poorly understood.

Recently, the GTPase RalA was found to be a key regulator of the secretory process of pancreatic β-cells [2]. However, in this study, neither the mechanisms leading to the activation of RalA in β-cells nor the precise events through which the GTPase controls the exocytotic process were determined. RalA and RalB share about 85% amino acid sequence identity and form a distinct subgroup of Ras-related monomeric GTPases. The two isoforms display a distinct tissue distribution and are involved in a variety of cellular processes including gene expression, cell migration, cell proliferation, oncogenic transformation and membrane trafficking [3], [4]. As is the case for other GTPases, activation of Ral proteins occurs via interaction with guanine nucleotide exchange factors (GEFs), which promote replacement of GDP for GTP. Many Ral-GEFs, such as RalGDS, Rlf/Rgl2, Rgl, RPM and Rgr, contain a Ras-binding domain and become activated upon interaction with the GTP-bound form of Ras [5], [6]. Ral proteins can also be stimulated by elevation of [Ca^2+^]_i_ through a Ras-independent mechanism [7]. In this case, Ral activation occurs via binding of the Ca^2+^ sensor calmodulin to the C-terminal domain of the GTPases [8]. Once activated, RalA and RalB accomplish their multiple functions by interacting with distinct downstream effectors [9]. Ral GTPases can control exocytosis by regulating the assembly of the exocyst [10], [11], a multiprotein complex initially identified in a genetic dissection of the yeast secretory pathway [12]. In mammals, the exocyst complex is required prior the formation of the SNARE complex and the fusion of secretory vesicles with the plasma membrane [13]. Assembly of the exocyst complex is required for the docking of insulin-containing secretory granules with the plasma membrane of β-cells [14]. An alternative mechanism through which Ral GTPases can affect vesicular transport is linked to their capacity to activate phospholipase D1 (PLD1), a key regulatory enzyme that plays an important role in membrane trafficking and cytoskeleton dynamics [15]. The formation of phosphatidic acid catalyzed by PLD1 is believed to favour the curvature of phospholipid membranes and to facilitate the fusion of secretory vesicles with the plasma membrane. Indeed, RalA-mediated activation of PLD1 was shown to be required for exocytosis of neuroendocrine cells [16]. Since PLD1 is known to play an important role in insulin secretion [17], [18], a similar mechanism could potentially underlie also the effect of RalA in pancreatic β-cells.

In this study, we used a combination of biochemical and imaging techniques to investigate the signalling pathways leading to RalA activation in pancreatic β-cells and to determine the mechanisms through which this GTPase controls insulin exocytosis. Our data show that RalA activation is mediated by the exchange factor RalGDS and is triggered by increases in [Ca^2+^]_i_ and cAMP. Silencing of RalA by RNA interference (RNAi) resulted in a decrease in insulin secretion that was associated with a reduced number of secretory granules docked at the plasma membrane and a diminished activation of PLD1 in response to secretagogues.

## Results

**Figure 1:**
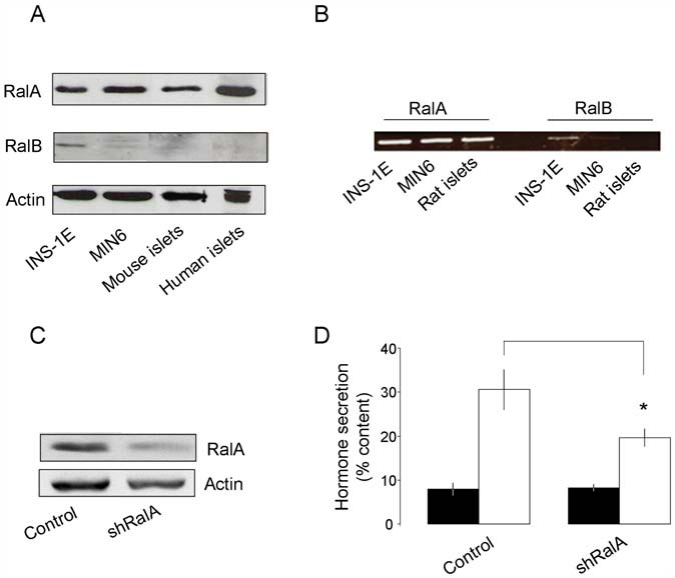
Expression of RalA in insulinoma cell lines and pancreatic islets and effect of RalA knockdown on hormone secretion. **A**) Aliquots (20 µg of proteins) of homogenates of INS-1E and MIN6 cell lines, as well as isolated mouse and human pancreatic islets were analyzed by Western blotting using antibodies against RalA, RalB or actin (internal control). **B**) Total RNAs extracted from INS-1E and MIN6 cell lines, as well as from freshly isolated rat pancreatic islets were analyzed by RT-PCR using primers specific for RalA or RalB. The PCR reactions yielded one single band of the expected size. **C**) INS-1E cells were transiently transfected either with an empty vector or with a plasmid driving the expression of a shRNA specifically directed against RalA (shRalA). Three days after transfection, the levels of the GTPase and of actin were assessed by Western blotting. **D**) INS-1E cells were transiently transfected with a plasmid encoding hGH (Control) or with a plasmid driving the expression of both hGH and shRalA. Three days after transfection, the cells were preincubated for 30 min under basal conditions (2 mM glucose), and then incubated for 45 min either under basal (filled bars) or stimulated conditions (20 mM glucose, 10 µM forskolin, 100 µM IBMX, open bars). hGH secretion was quantified by ELISA. The results are expressed as hGH secretion under basal and stimulatory conditions (normalized to the content) and represent the mean ± SEM of six independent experiments. * p<0.05 ANOVA.

We first determined which Ral isoform is expressed in pancreatic β-cells. RalA was readily detected in the insulin-secreting cell lines INS-1E and MIN6 as well as in mouse and human pancreatic islets (Fig. 1A). INS-1E cells and, to a much lesser extent, MIN6 cells were found to express small amounts of RalB but this isoform was undetectable in mouse and human pancreatic islets. These findings are unlikely to reflect differences in the affinities between RalA and RalB antibodies. Indeed, the RalB antibody readily detected the protein in insulin-secreting cells overexpressing the GTPases (Figure S1). Moreover, RalA and RalB mRNA levels assessed by RT-PCR analysis mirrored the differences observed by Western blotting and showed that RalA is also the predominant isoform in rat pancreatic islets (Fig. 1B). In view of these findings, the following experiments focussed on the role of the RalA isoform.

Recently, RalA has been demonstrated to be required for the regulation of insulin secretion [2]. To investigate the involvement of RalA in β-cell exocytosis, INS-1E cells were transfected with a plasmid driving the synthesis of both a small hairpin RNA (shRNA) directed against the GTPase and of human growth hormone (hGH) [18]. When expressed in β-cells hGH is targeted to secretory granules and is co-released with insulin. This allowed us to monitor exocytosis specifically in the fraction of cells expressing the silencer. As shown in Fig. 1C, transfection of the shRNA resulted in a decrease in the level of RalA. Western blot quantification revealed that RalA level was reduced by about 50% in cells expressing the RalA shRNA. Silencing of the GTPase was accompanied with a reduction of 40% in hormone secretion elicited by glucose and cAMP-raising agents (p<0.05, n = 7) compared to cells transfected with an empty vector or to cells expressing a shRNA against GFP (Fig. 1D; Figure S2). Basal secretion measured in the presence of 2 mM glucose was unaffected. These observations are in good agreement with the findings of Lopez et al. [2], and confirm that RalA is required to maintain optimal secretory functions in pancreatic β-cell.

**Figure 2:**
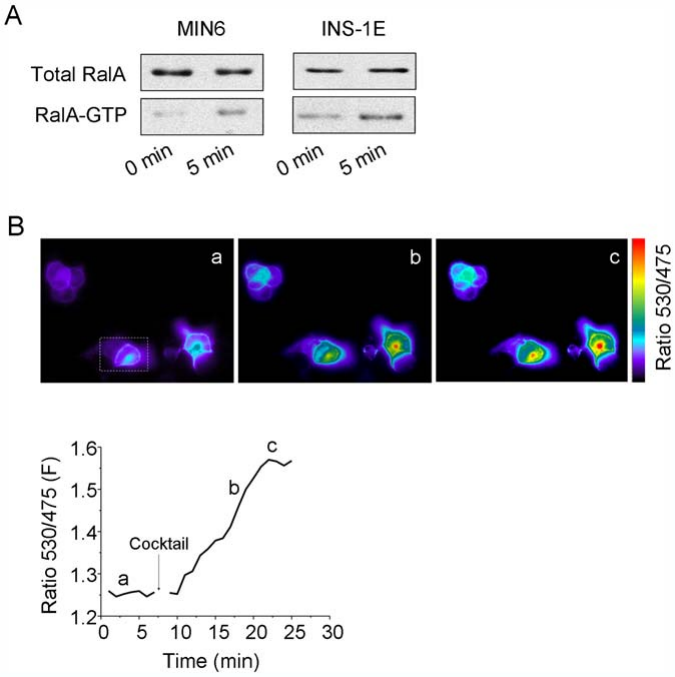
Kinetics of RalA activation in response to secretagogues. **A**) INS-1E and MIN6 cells were first preincubated at 2 mM glucose for 30 min and then stimulated by addition of a medium containing 20 mM glucose, 30 mM KCl, 10 µM Forskolin and 100 µM IBMX. After 5 min, the cells were lysed and the GTP-bound form of RalA isolated on GST-affinity columns. The total amount of RalA present in the cell lysates and the fraction of the GTPase remaining associated with the beads were visualized by Western blotting. The figures show blots from a representative experiment out of three. **B**) To monitor RalA activation in real-time, INS-1E cells were transfected with the pRaichu-RalA probe [20]. Two days later the cells were first incubated at basal condition and then stimulated (arrow) with a cocktail of secretagogues including 20 mM glucose, 30 mM KCl, 10 µM Forskolin and 100 µM IBMX. RalA activation was monitored in real-time by measuring the intensity of the fluorescence at 530 and 475 nm and by determining the changes in the FRET ratio (530 nm/475 nm emissions). The upper images show the evolution of the FRET ratio in a group of cells after the addition of the secretagogues. Image a: 0 min; Image b: 10 min; Image c: 15 min. The lower panel shows the curve corresponding to the kinetics of RalA activation in the cell surrounded by the square.

**Figure 3:**
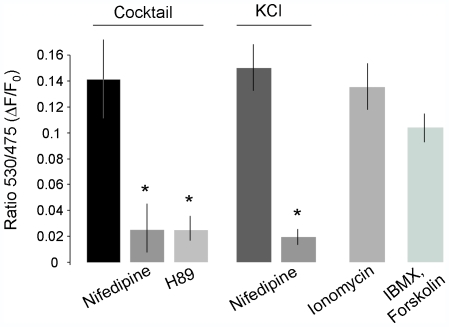
RalA activation is triggered by increases in [Ca^2+^]_i_ and cAMP. The signaling pathways leading to RalA activation were explored by transfecting INS-1E cells with the pRaichu-RalA probe [20] and by analyzing the fluorescence emissions at 530 nm and 475 nm. The results are expressed as maximum changes in the 530 nm/475 nm ratio (ΔF) divided by the value of the ratio at the beginning of the experiment (F_0_). The cells were stimulated with a combination of secretagogues (Cocktail) containing 20 mM glucose, 30 mM KCl, 10 µM Forskolin and 100 µM IBMX (n = 11 cells), with 30 mM KCl (n = 6 cells), with 1 µM of Ionomycin (n = 9 cells) or with 10 µM Forskolin and 100 µM IBMX (n = 9 cells). Where indicated the cells treated with the cocktail of secretagogues were preincubated for 15 min with 10 µM of the voltage-gated Ca^2+^ channel blocker Nifedipine (n = 9 cells) or with 10 µM of the PKA inhibitor H89 (n = 6 cells). A group of cells stimulated with 30 mM KCl were also treated with 10 µM Nifedipine (n = 6 cells). * Denotes the conditions significantly different (p<0.05 ANOVA test) from the respective controls.

In the next set of experiments we investigated the mechanisms leading to activation of RalA in response to different substances capable of triggering insulin release. We initially assessed the activation of the GTPase by capturing the activated form of RalA on a GST-affinity column containing a fragment of Ral binding protein 1 (RalBP1) [19]. Using this approach, we found that incubation of MIN6 or INS-1E cells for 5 min in the presence of 20 mM glucose, 10 µM Forskolin, 100 µM IBMX and 30 mM KCl lead to a 2–3 fold increase in the amount of RalA in the GTP-bound form (n = 3, Fig. 2A). To investigate the kinetics of RalA activation, we then took advantage of a recently developed Fluorescence Resonance Energy Transfer (FRET)-based probe for RalA activity, called pRaichu-RalA [20]. The pRaichu-RalA construct encodes a chimeric fluorescent protein that includes from the N-terminus: a modified form of YFP, wild type RalA, the Ral binding domain of RalBP1 and a modified form of CFP [20]. When RalA is activated, the GTPase binds to the RalBP1 fragment and triggers a conformational change in the molecule that enables a FRET [21] between the two fluorophores, CFP and YFP. Thus, in INS-1E cells transfected with the pRaichu-RalA construct the activation of RalA can be monitored in real-time by analysing the ratio between the emissions of YFP and CFP. The probe displayed a subcellular distribution analogous to that of GFP-tagged RalA (Figure S3). The chimeric construct was detectable throughout the cytoplasm but was particularly abundant near the plasma membrane region. As shown in Fig. 2B, incubation of the cells in the presence of the cocktail of secretagogues including 20 mM glucose, 10 µM Forskolin, 100 µM IBMX and 30 mM KCl led to a strong rise in the FRET ratio. The increase in FRET was detectable after a lag of 3–5 min and reached a plateau between 10–15 min. The activation of RalA observed in the presence of the secretagogues was reduced by more than 80% by the L-type voltage-gated Ca^2+^-channel blocker Nifedipine (10 µM), suggesting that the activation of the GTPase necessitates an increase in [Ca^2+^]_i_ (Fig. 3). In agreement with this hypothesis, a rise in [Ca^2+^]_i_ elicited by incubation of INS-1E cells with depolarizing K^+^ concentrations (30 mM) or with the Ca^2+^ ionophore Ionomycin (1 µM) resulted in a comparable activation of RalA (Fig. 3). Part of the effect of the secretagogues may also be linked to a rise in cAMP levels. Indeed, the activation of RalA was diminished by approximately 80% by treatment of the cells with the protein kinase A inhibitor H89 (10 µM). Moreover, it was possible to activate the GTPase with the cAMP-raising agents Forskolin (10 µM) and IBMX (100 µM) even in the presence of low glucose concentrations (Fig. 3).

**Figure 4:**
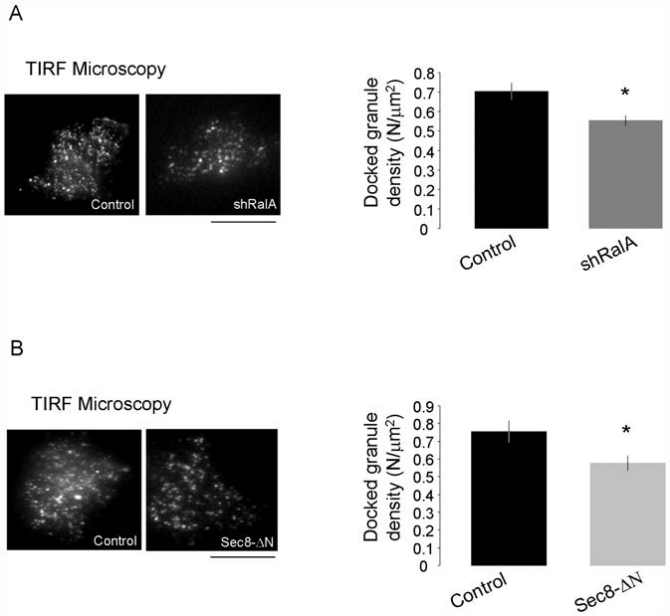
Effect of RalA silencing on insulin granule docking. **A**) Experiments were performed under TIRF illumination. INS-1E cells were co-transfected with a plasmid encoding IAPP-EGFP (a fluorescently labeled protein targeted to insulin granules) and either an empty vector (control) or shRalA. On the left: representative TIRF images of control and shRalA transfected cells. On the right: histograms depict the number of granules/µm^2^ counted in the “footprint” of each cell using TIRF illumination in control conditions and in cells transfected with shRalA. Results are the means ± SEM (n = 23 cells control; n = 40 cells shRalA). * p<0.05 one way ANOVA. Scale bar: 12 µm **B**) INS-1E cells were co-transfected with a plasmid encoding NPY-mRFP (a fluorescently labeled protein targeted to insulin granules) and either an empty vector (control) or a plasmid driving the expression of a dominant-negative form of Sec8 (Sec8-ΔΝ). Quantitative PCR analysis confirmed that in transfected cells the dominant-negative transcript was approximately 1000-folds more abundant than the endogenous Sec8 mRNA. On the left: representative TIRF images of control and Sec8-ΔΝ transfected cells. On the right: histograms represent number of granules/µm^2^ counted in the “footprint” of each cell under TIRF illumination in control conditions and in cells transfected with ΔSec8. Results are mean ± SEM (n = 22 cells control; n = 29 cells, Sec8-ΔΝ). * p<0.05 one-way ANOVA. Scale bar: 12 µm.

The next set of experiments aimed at determining the mechanisms through which RalA controls insulin exocytosis. Ral GTPases are thought to participate to vesicle docking by modulating the assembly of the exocyst complex [12]. To assess whether RalA is necessary for the docking of insulin-containing granules, INS-1E cells were transfected with a plasmid encoding fluorescently labelled Islet Amyloid Precursor Protein (IAPP-EGFP). This protein is targeted to insulin-containing granules and can be used to visualize the secretory vesicles of β-cells [22]. To determine the number of secretory granules docked at the plasma membrane, IAPP-EGFP-transfected cells were inspected by total internal reflection fluorescence (TIRF) illumination [23]. Using this technique, only fluorescently labelled vesicles located within about 90 nm from the plasma membrane are detected [24], [25], [26]. We found that silencing of RalA reduced the number of docked granules by about 22% (n = 24 cells, control; n = 40 cells. shRalA; *p<0.05) (Fig. 4A). This effect could not be ascribed to a reduced overall number of granules in the presence of shRalA because the total IAPP-EGFP fluorescence/cell under epifluorescence illumination was identical in shRalA expressing cells and in control conditions (control cells: 141.6±4.31, n = 45; shRalA 140.317±5.17, n = 45; Sec8-ΔΝ; 132.35±8.34, n = 18). The effect of shRNA against RalA on docking of insulin-containing granules was confirmed using a shRNA against GFP as a second control (Figure S2). Interestingly, the impact of RalA knockdown on granule docking was comparable to the effect obtained after transfection of INS-1E cells with a dominant-negative truncated form of the exocyst component Sec8 (n = 22 cells, control; n = 29 cells, Sec8-ΔΝ; * p<0.05, Fig. 4B), a construct that has previously been shown to inhibit granule docking and exocytosis in β-cells [14].

**Figure 5:**
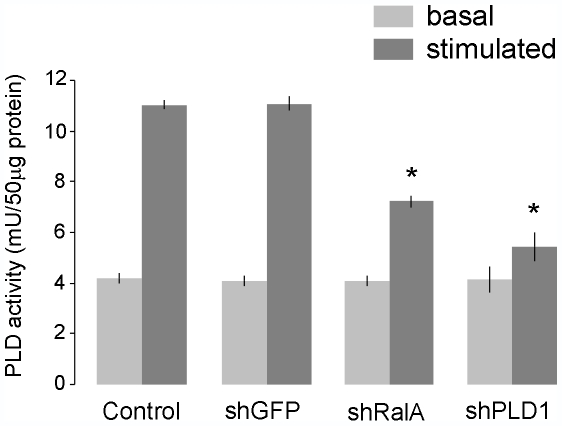
Effect of RalA silencing on PLD activity. INS-1E cells were transfected with an empty vector (Control), with a plasmid encoding a shRNA directed against GFP (shGFP), RalA (shRalA) or PLD1 (shPLD1) [18]. Three days later the cells were incubated under basal conditions in a medium containing 2 mM glucose or were stimulated during 10 min with 20 mM glucose, 30 mM KCl, 10 µM Forskolin and 100 µM IBMX. At the end of the incubation period, cell lysates were prepared and assayed for PLD activity. Histograms show PLD activity (mU/50 µg of proteins) measured under basal and stimulatory conditions. The results are the means ± SD of three independent experiments. * p<0.05, one-way ANOVA.

The N-terminal domain of RalA can bind and activate PLD1, a key player in the final events leading to insulin exocytosis [17], [18]. To determine the contribution of RalA to PLD1 activation in β-cells, we measured the PLD activity in INS-1E cells upon silencing of the GTPase. We found that basal activity that is mainly contributed by PLD2 was unaffected. In contrast, secretagogue-induced PLD activation that reflects PLD1 activity was strongly reduced in cells lacking RalA (Fig. 5) (n = 3, * p<0.05). The effect of RalA silencing was comparable to the decrease in PLD activity observed after silencing of PLD1 by RNAi [18]. Taken together, these observations indicate that the control of PLD1 activation constitutes an additional mechanism through which RalA can modulate the secretory process of β-cells.

**Figure 6:**
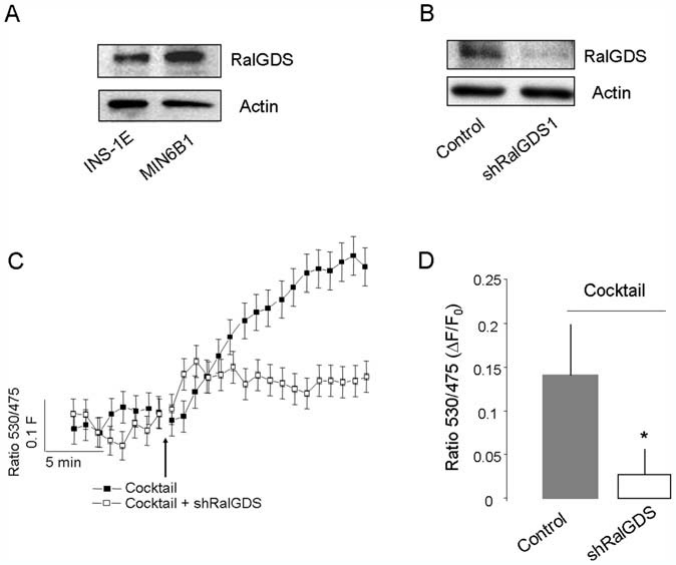
Silencing of RalGDS prevents the activation of RalA. **A**) Expression of RalGDS in INS-1E and MIN6 cells assessed by Western Blotting. **B**) The efficiency of a shRNA directed against RalGDS (shRalGDS1) was verified by transiently co-transfecting INS-1E cells with the shRNA-expressing vector. The level of the exchange factor was analyzed two days later by Western blotting. The figure shows a representative experiment out of three. **C**) To observe the effect of RalGDS knockdown on RalA activation, INS-1E cells were co-transfected with pRaichu-RalA and either an empty vector (filled squares) or with shRalGDS1 (open squares). Two days later, the cells were stimulated (arrow) with a cocktail of secretagogues containing 20 mM glucose, 30 mM KCl, 10 µM Forskolin and 100 µM IBMX and RalA activity was followed in real-time. The figure shows a representative experiment with the mean ± SD of the variations in the 530 nm/475 nm ratio of three control cells and three cells transfected with shRalGDS1. **D**) Quantification of RalA activation was performed by monitoring the variations between the fluorescence at 530 nm and 475 nm in cells transfected with an empty vector (Control) and cells expressing shRalGDS1. The results are expressed as maximum changes in the 530 nm/475 nm ratio (ΔF) divided by the value of the ratio at the beginning of the experiment (F_0_) ± SD (Control, n = 7 cells; shRalGDS, n = 10 cells). * p<0.05 ANOVA.

**Figure 7:**
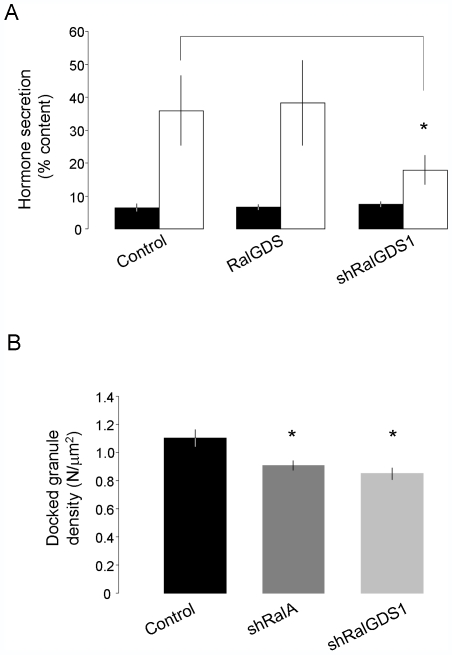
Knockdown of RalGDS leads to inhibition of hormone secretion and granule docking. **A**) To evaluate the involvement of RalGDS in the control of hormone secretion, INS-1E cells were co-transfected with a plasmid encoding hGH and with an empty vector (Control), a plasmid permitting the overexpression of RalGDS or with shRalGDS1. Three days later the cells were incubated for 30 min at basal conditions (2 mM glucose), and then 45 min either at basal (filled bars) or stimulated conditions (20 mM glucose, 10 µM Forskolin and 100 µM IBMX, open bars). Hormone release was quantified by ELISA. The histograms represent the means ± SEM of three independent experiments. * Significantly different (p<0.05 ANOVA). **B**) INS-1E cells were co-transfected with a plasmid encoding IAPP-EGFP and either an empty vector (Control), shRalA or shRalGDS1. Histograms show the number of granules/µm^2^ counted in the “footprint” of each cell by using TIRF illumination in control conditions and in cells transfected with shRalA. Results are expressed as means ± SEM (n = 24 cells control; n = 36 cells shRalA; n = 27 cells shRalGDS1). * p<0.025 Bonferroni test.

Activation of Ral proteins occurs via interaction with guanine nucleotide exchange factors (GEFs), which promote replacement of GDP for GTP [5], [6]. The GDP/GTP exchange factor RalGDS has recently been shown to activate Ral proteins in response to [Ca^2+^]_i_ and cAMP elevations in endothelial cells [27]. This exchange factor was found to be expressed both in MIN6 and INS-1E cells (Fig. 6A). To investigate the possible involvement of RalGDS in the regulation of RalA activity in β-cell, we first generated a plasmid encoding a shRNA specifically directed against RalGDS (shRalGDS1). We found that, when introduced in INS-1E cells, the silencer led to a strong decrease of RalGDS mRNA (Figure S4) and reduced by about 60% the expression of the endogenous RalGDS protein (Fig. 6B). The impact of RalGDS silencing on RalA activation was then investigated by FRET analysis. RalA activation triggered by secretagogues was found to be severely impaired in INS-1E cells co-transfected with pRaichu-RalA and shRalGDS1 (Fig. 6C and D). Analogous results were obtained when the activation of RalA was assessed using GST-Pull down experiments (not shown). We then tested the effect of RalGDS silencing on secretagogue-induced hormone release (Fig. 7A). Our results show that hormone secretion is diminished by more than 30% in cells lacking the exchange factor (p<0.05, n = 3), whereas overexpression of RalGDS did not further increase hormone release. This effect on secretion was accompanied with a 24% decrease in the number of granules docked at the plasma membrane, a reduction comparable to that obtained upon silencing of RalA (Fig. 7B). Similar results were obtained using a different shRNA (shRalGDS2) directed against RalGDS (Figure S4, S5 and S6). Together, these findings indicate that RalGDS plays a major role in RalA activation in response to insulin secretagogues.

## Discussion

Ral GTPases are multifunctional proteins that regulate a variety of cellular processes including oncogenic transformation, cell proliferation and vesicular trafficking [3], [4]. Although RalA and RalB share about 85% of their amino acid sequences the functions of the two isoforms are only partially overlapping. Indeed, RalA and RalB have antagonistic effects on cancer cell migration [28] and RalA but not RalB drives delivery of membrane proteins to the basolateral surface of epithelial cells [29]. Here, we found that RalA is the predominant isoform expressed in insulin-secreting cell lines and in pancreatic islets of different species. Low levels of RalB were detected in insulinoma cell lines but not in freshly isolated pancreatic islets and may be the consequence of oncogenic transformation. For this reason, in this study the function of RalB was not investigated and for the moment the role, if any, of this isoform in β-cells remains unsettled.

Ral GTPases are involved in the regulation of exocytosis in different cell systems [4]. Recently, RalA was found to be an important modulator of the secretory process of pancreatic β-cells [2]. Here, we have first confirmed the involvement of RalA in insulin exocytosis and we have then investigated the signalling cascades leading to the activation of the GTPase and the mechanisms through which it controls hormone release. We have monitored RalA activation in the presence of insulin secretagogues using imaging techniques that allow a good spatio-temporal resolution of the generation of the active form of the GTPase. The appearance of the GTP-bound form of RalA started few minutes after the addition of the stimuli and peaked in about 10–15 minutes, consistent with the time course of insulin exocytosis. Under our experimental conditions we did not observe preferential activation of RalA in specific subcellular compartments. This could be the consequence of the type of stimuli used in this study and spatial differences may possibly be observed upon incubation with agonists that lead to second messenger generation in restricted areas of the cells.

Two second messenger cascades were found to contribute to RalA activation: one triggered by an increase in [Ca^2+^]_i_, resulting from entry of the ion through L-type voltage-gated Ca^2+^ channels, and the other by elevation of cAMP. Activation of RalA via these signalling pathways has been reported in other cell types [27]. The stimulatory effect of Ca^2+^ could potentially be mediated through direct interaction of Ca^2+^/CaM to the C-terminus of RalA [8], [30] and/or through binding of Ca^2+^/CaM to RalGDS with a consequent stimulation of the exchange factor function [27]. RNAi experiments performed in INS-1E cells revealed that silencing of RalGDS lead to an almost complete blockade of RalA activation, suggesting that in β-cells the elevation of [Ca^2+^]_i_ stimulates the GTPase mainly via the second mechanism. The generation of cAMP in pancreatic β-cells triggers two parallel signalling pathways regulating insulin exocytosis: one involving PKA activation and the other the activation of Epac2 (Exchange proteins directly activated by cAMP 2) [31]. We found that the appearance of the GTP-bound form of RalA is prevented in INS-1E cells treated with H89, indicating that activation of RalA occurs via the PKA-dependent pathway. Interestingly, RalGDS is known to be phosphorylated and activated by PKA. Thus, the exchange factor RalGDS that is positioned at the “crossroad” of the two signalling pathways appears to be the central regulator of RalA function in β-cells.

The goal of the next series of experiments was to determine the precise mechanism(s) through which RalA can modulate the secretory process of β-cells. Ral GTPases have been shown to control the association of the components of the exocyst [10], [11]. This octameric protein complex is required for exocytosis by tethering vesicles to specific sites at the plasma membrane before the formation of the SNARE fusion complex [12]. In pancreatic β-cells, impairment in the assembly of the exocyst results in a decrease in the number of secretory granules docked at the plasma membrane and in defective insulin release [14]. We found that silencing of RalA in INS-1E cells lead to a reduction in the number of granules docked at the plasma membrane. The impact of RalA knockdown on granule docking was comparable to that obtained after introduction of a dominant negative Sec mutant that prevents the assembly of the exocyst complex [14]. These observations suggest that part of the effect of RalA on β-cell exocytosis may be exerted through the regulation of the assembly of the exocyst components. Using an electron microscopy approach, no significant changes were detected in the number of docked granules in PC12 cells lacking both RalA and RalB [32]. At present, it is unclear whether the discrepancy between our findings and the results of this study resides in peculiar differences in the two cell systems or in the sensitivity of the techniques used to determine the number of docked granules.

A relatively limited decrease in the pool of granules docked at the plasma membrane is not necessarily associated with a defect in exocytosis. We therefore investigated whether RalA is also involved in the control of other important steps of the secretory pathway. We have previously demonstrated that in chromaffin and PC12 cells RalA is necessary for the activation of PLD1 together with the GTPase ARF6 [16]. This phospholipase plays an essential role in insulin exocytosis by catalysing the formation of the fusogenic lipid phosphatidic acid [17], [18]. Interestingly, ARF6 was also shown to play an important regulatory function in insulin release [33]. Here, we show that the burst in PLD1 activity triggered by insulin secretagogues is strongly reduced in INS-1E cells lacking RalA. These observations identify the modulation of an ARF6-dependent PLD1 activity as an additional mechanism through which RalA can control insulin exocytosis.

In this study, we have dissected the signalling cascades leading to RalA activation in pancreatic β-cells and we have demonstrated the involvement of this GTPase in two distal steps of the insulin secretory pathway. RalA is a multifunctional protein that via its large number of interacting partners controls other cellular events potentially affecting the execution of the secretory process. Thus, the capacity of RalA to influence the dynamics of the actin cytoskeleton and to modulate endocytosis could constitute additional means to finely tune insulin secretion. Future experiments will have to assess whether the control exerted by RalA on the secretory process of β-cells extends beyond the mechanisms identified in this study. Moreover, in addition to its role in the regulation of insulin release it will be important to define the possible involvement of RalA in other β-cell functions that when deregulated favour the development of diabetes mellitus such as apoptosis or proliferation.

## Materials and Methods

### Ethics statement

Rat and mouse islets were isolated by hand-picking after collagenase digestion of pancreas as described [36]. The isolation protocol was approved by the animal care committee of the Veterinary Office of Canton Vaud. Human islets were provided through the European Consortium for Islet Transplantation (ECIT), supported by JDRF grant N° 31-2008-416. The use of human islets for research was approved by the Geneva local institutional ethical committee.

### Cell culture

The rat INS-1E insulinoma cell line [34] was cultured in RPMI 1640 supplemented with 10% fetal calf serum, 70 µM β-Mercaptoethanol, 50 units/ml penicillin, 50 µg/ml streptomycin and 0.1 mM sodium pyruvate. Mouse MIN6B1 insulinoma cells [35] were grown in Dulbecco's modified Eagle's medium supplemented with 15% fetal calf serum, 70 µM β-Mercaptoethanol, 50 units/ml penicillin and 50 µg/ml streptomycin.

### Plasmids and transfection

The pRaichu-RalA probe [20] used for FRET imaging was generously provided by Dr. M. Matsuda, Kyoto University, Japan. The mammalian expression vector encoding mouse RalGDS was a gift from Dr. A. Wittinghofer, Max-Planck Institute, Leipzig, Germany. The plasmid encoding EGFP-tagged N-terminally truncated Sec8 (pIRES2- Sec8-ΔΝ, called here Sec8-ΔΝ) was obtained from Dr. G. Rutter, Imperial College of London, UK. The shRNA against rat RalA (shRalA) and the two shRNAs against rat RalGDS (shRalGDS1 and shRalGDS2), targeting the nucleotide sequences AAGGCAGGTTTCTGTAGAA, GCCATGGACAAACACAACC and CTCTGCCGCCAACTATGAC, respectively, were prepared by inserting the corresponding oligonucleotides in the Hind III/Bgl II sites of a modified pSuper vector engineered to express the human Growth Hormone (hGH) [16]. The shRNA against PLD1 has been described previously [18]. Control cells were either transfected with an empty plasmid or with a pSuper plasmid expressing a shRNA targeting the GFP sequence GGCTACGTCCAGGAGCGCA (shGFP). Transient transfection experiments were performed as described [37] using Lipofectamine 2000 (Invitrogen. Carlsbad, CA). Under our experimental conditions, the transfection efficiency evaluated by fluorescence activated cell sorting of EGFP-transfected cells is about 55%.

### Immunoblotting

INS-1E cells, MIN6B1 cells or purified pancreatic islets were resuspended in PBS and briefly sonicated. The homogenates were centrifuged at 4°C for 1 minute at 10’000 x g to pellet nuclei and cell debris. 30 µg of proteins collected in the supernatant were separated by SDS-PAGE, transferred to nitrocellulose membranes and detected using specific antibodies. The RalA antibody was obtained from BD Transductions Laboratories (Lexington, KY), RalB and RalGDS antibodies were purchased from Santa Cruz Biotechnology (Santa Cruz, CA) and the β-actin antibody from Sigma (St. Louis, MO). Visualization of the immunocomplexes was performed by chemiluminescence (Amersham Biosciences, Piscataway, NJ) after incubation of the nitrocellulose membranes with secondary antibodies coupled to horse radish peroxidase.

### RT-PCR Analysis

Total RNA was prepared using the Ambion's RNA extraction kit (Austin, TX). Reverse transcription reactions were performed as previously described [38]. For RalA and RalB mRNA expression studies 30 amplification cycles were carried out in the presence of the following primers: RalA sense, 5′-ACAAGCCCAAGGGTCAGAAT-3′; antisense, 3′-ACTGCCCACCATGATGACTT-5′; RalB sense, 5′-GGCCATCCGTGACAACTACT-3′; antisense, 3′-GAGAACACCAGCAGAAAGC-5′. RalGDS and Sec8 mRNA levels were assessed by quantitative real-time PCR (qRT-PCR) analysis carried out on a BioRad MyIQ Single-Color Real-Time PCR detection system using the following primers: RalGDS sense, 5′-ACTCCCTGAGCAGAGAGCTG-3′; RalGDS antisense, 3′-GGGCTCTCCTAGGGTTCATC-5′; Sec8 sense, 5′-GCCGAACAAAGTCAGCTTTC-3′; Sec8 antisense, 5′-CACTTCCAGATGCAAGACGA-3′. Normalization of the qRT-PCR reaction was performed by measuring GAPDH mRNA levels with the following primers: GAPDH sense, 5′-TCCATGACAACTTTGGCATTG-3′; GAPDH antisense, 5′CAGTCTTCTGGGTGGCAGTGA-3′.

### Secretion assay

Secretion experiments in INS-1E cells were performed three days after transfection. The cells were washed and then preincubated in a KREBS-Ringer/bicarbonate-Hepes buffer (KRBH: 127 mM NaCl, 4.7 mM KCl, 1.2 mM KH_2_PO_4_, 1.2 mM MgSO_4_, 1 mM CaCl_2_, 5 mM NaHCO_3_, 25 mM Hepes pH 7.4 and 0.1% bovine serum albumin) containing 2 mM glucose. After 30 min at 37°C, the buffer was discarded and the cells incubated either again in KRBH containing 2 mM glucose (basal condition) or with KRBH containing 20 mM glucose and supplemented with 10 µM Forskolin and 100 µM IBMX (stimulatory condition). The amount of hGH released into the medium under basal and stimulatory conditions and the fraction of hormone remaining in the cells at the end of the incubation period were quantified by ELISA (Roche Diagnostics, Rotkreuz, CH).

### Measurement of RalA activation using a pull-down assay

MIN6B1 or INS-1E cells cultured in 6-well plates were preincubated at 37°C for 30 min under basal conditions (KRBH containing 2 mM glucose). The medium was then removed and the cells incubated for 5 min in KRBH containing 2 mM glucose or in KRBH containing 20 mM Glucose, 10 µM Forskolin, 100 µM IBMX and 30 mM KCl. The cells were then lysed in ice cold pull-down buffer (50 mM Tris-HCl, pH 7.5, 200 mM NaCl, 1% NP-40, 10 mM MgCl_2_, 0.5 mM dithiothreitol, 1 mM PMSF and 10 µg aprotinin). The lysates were cleared by spinning at top speed for 10 min on a bench-top centrifuge and then passing through a 0.22 µm filter. Aliquots of 250–500 µg of protein lysate were mixed with GST-RalBP1 agarose beads (Upstate Biotechnology, Waltham, MA) and incubated for 30–60 min at 4°C. The beads were washed three times in pull-down buffer and the proteins remaining associated with the beads resuspended in Laemmli buffer. The fraction of GTP-bound RalA recovered on the GST-affinity column was determined by Western blotting.

### Measurement of RalA activity in living cells with Fluorescence resonance energy transfer (FRET) imaging

Real-time assessment of RalA activity was carried out by using the Raichu-RalA probe [20]. INS-1E cells plated on glass coverslips coated with 2 mg/ml poly-L-lysine and 33 µg/ml laminin were co-transfected with the pRaichu-RalA probe and with the indicated plasmids. Two days after transfection, the cells were preincubated at low glucose for 30 min. The coverslips were then mounted in an open perfusion incubator at 37°C on the stage of a Zeiss Axiovert 200 fluorescence inverted microscope modified for imaging experiments [39]. The cells were imaged through a 63 x oil immersion objective (Zeiss, Germany) at 1 min intervals with a cooled charged-coupled device (SNAP-HQ CCD) camera. Excitation of the samples at 433 nm was obtained with a polychromator lamp (Visitron System, Germany). Emissions at 530 and 475 nm were collected with two cube filter sets: 51017 filter set for CFP and 41028 filter set for YFP (Omega Optical, Brattleboro, VT). The camera and the polychromator were controlled by the Metafluor software (Universal Imaging, Ypsilanti MI). For analysis, the 530 and 475 nm emissions were monitored in ROIs centered on top of each cell. After background subtraction the FRET ratio (530/475 nm) was calculated with Origin software (Northampton, MA).

### Imaging docked granules with Total Internal Fluorescence Reflection (TIRF) microscopy

For TIRF experiments, INS-1E cells were plated on glass coverslips coated with 2 mg/ml poly-L-lysine and 33 µg/ml laminin. Insulin-containing secretory granules were imaged by transfecting the cells either with IAPP-Emerald [22] or with NPY-mRFP [40]. Two days after transfection the cells were analysed using a Zeiss Axiovert 200 inverted fluorescence microscope modified to allow epifluorescence and evanescence field (TIRF) illumination [24], [25], [26]. The cells were incubated under resting conditions during the whole experiment. For TIRF illumination, the expanded beam (488 nm–568 nm argon krypton multi-line laser, 20 mW) was passed through an AOTF wavelength selector synchronized with a SNAP-HQ CCD Camera under Metafluor software control and introduced from the high numerical aperture objective lens (100 X, 1.45 NA, Zeiss, Germany). Light entering the coverslips underwent total internal reflection at the glass-cell interface with a penetration depth of about 100 nm. Light were filtered with a beam splitter (Zeiss filter set 10). Video images were digitized and analyzed using the Metamorph software (Universal Imaging, Ypsilanti MI). Single IAPP-Emerald or NPY-mRFP positive granules were counted manually in single cell areas. Only cells displaying a docking surface area of at least 100 µm^2^ were selected. Granule densities (number of docked granules per µm^2^) under the different experimental conditions were compared using the analysis of variance (GLM procedure from SAS statistical package, SAS Institute Inc., Cary, NC, USA), with the multiple comparisons of means (Bonferroni post hoc test).

### Measurement of PLD activity

INS-1E cells cultured in 6-well plates to about 80% confluency were washed twice and preincubated in KRBH containing 2 mM glucose during 30 min. The medium was then removed and the cells were either incubated again in the same buffer or stimulated for 10 min in KRBH containing 20 mM glucose, 10 µl Forskolin, 100 µl IBMX30 and mM KCl. At the end of the incubation period the cells were resuspended in ice-cold Tris-HCl (50 mM, pH 8.0) and disrupted by three freeze/thawing cycles. PLD activity was measured in 50 µg of cell extracts using the Amplex Red phospholipase D assay kit (Invitrogen Corporation, Carlsbad CA) according to the manufacturer's instructions. A standard curve for PLD activity was obtained by incubating under the same conditions aliquots of purified PLD from *Streptomyces chromofuscus* (Sigma, St. Louis MO).

### Statistical analysis

The experiments were analyzed using the SAS statistical package (SAS Inc., Cary NC, USA). The analysis of variance (ANOVA) procedure was used followed, when indicated, by the Bonferroni multiple comparisons post-hoc test with a significance limit set at p<0.05.

## Supporting Information

Figure S1 Detection of Ral GTPases in INS-1E cells overexpressing RalA or RalB. Samples obtained from INS-1E cells transfected with an empty vector (control) and from cells transfected with RalA or RalB expressing plasmids were analyzed by Western blotting using antibodies against RalA (A) or RalB (B). Equal loading between the different lanes was assessed by analyzing the same samples with an antibody against actin.
(0.70 MB TIF)

Figure S2 Comparison between the effect of shRalA and of a shRNA against GFP on hormone secretion and granule docking. A) INS-1E cells were co-transfected with a plasmid encoding hGH and with plasmids permitting the expression of shGFP or shRalA. Three days later the cells were incubated for 30 min at basal conditions (2 mM glucose), and then 45 min either at basal (filled bars) or stimulated conditions (20 mM glucose, 10 µM Forskolin and 100 mM IBMX, open bars). Hormone release was quantified by ELISA. The results are the means ± SEM of five independent experiments. * p<0.05 ANOVA. B) INS-1E cells were co-transfected with a plasmid encoding NPY-mRFP and either shGFP or shRalA. The number of granules docked at the plasma membrane per µm^2^ was observed and counted by inspecting the cells by TIRF microscopy. Silencing of RalA resulted in a significant reduction in the number of docked granules per µm^2^ (Bonferroni test: * p<0.05). Results are presented as means ± SEM (n = 23 cells, shGFP; n = 22 cells, shRalA).
(0.46 MB TIF)

Figure S3 Subcellular localization of the chimeric probe pRaichu-RalA. INS-1E cells transfected with the pRaichu-RalA plasmid (A) or with GFP-tagged RalA (B) were analyzed by confocal microscopy. Notice that both fluorescent probes display a similar subcellular distribution in the cytosolic and plasma membrane compartments. Scale bar 25 µm.
(1.19 MB TIF)

Figure S4 Effect of two different shRNAs on RalGDS mRNA level. To test the silencing efficiency of two shRNAs directed against RalGDS, total RNAs extracted from INS-1E cells expressing shGFP, shRalGDS1 or shRalGDS2 were analyzed by quantitative Real-Time PCR. Transfection of shRalGDS1 and shRalGDS2 reduced RalGDS mRNA levels by 83 and 85%, respectively. The results represent the means ± SD of three independent experiments. * p<0.001 ANOVA.
(0.43 MB TIF)

Figure S5 Effect of shRalGDS 1 and 2 on RalA activation. Quantification of RalA activation was performed by monitoring the variations between the fluorescence at 530 nm and 475 nm in cells transfected with shGFP, with shRalGDS1 or shRalGDS2. The results are expressed as maximum changes in the 530 nm/475 nm ratio (ΔF) divided by the value of the ratio at the beginning of the experiment (F0) ± SD (n = 6 cells shGFP; n = 4 cells shRalGDS1; n = 4 cells shRalGDS2). * p<0.05, ANOVA.
(0.45 MB TIF)

Figure S6 Effect of shRalGDS 1 and 2 on hormone secretion and insulin granule docking. A) INS-1E cells were co-transfected with a plasmid encoding hGH and with shGFP, shRalGDS 1 or shRalGDS 2 encoding vectors. Hormone release under basal (filled bars) and stimulatory conditions (open bars) was assessed three days later by ELISA. The results are means ± SEM of six independent experiments. * p<0.05, ANOVA. B) INS-1E cells were co-transfected with a plasmid encoding NPY-mRFP and either shGFP, shRalGDS1 or shRalGDS 2. The granules docked at the plasma membrane were visualized by TIRF microscopy. Silencing of RalGDS with shRalGDS 1 or shRalGDS 2 resulted in a significant reduction in the number of docked granules per µm^2^ (Bonferroni test: * p<0.05). The results are given as means ± SEM (n = 23 cells, shGFP; n = 21 cells, shRalGDS 1; n = 22 cells, shRalGDS 2).
(0.50 MB TIF)
