# Supplementary figures and images for: The GTPase RalA Regulates Different Steps of the Secretory Process in Pancreatic β-Cells
Source: PLoS One. 2009 Nov 5;4(11):e7770. doi: 10.1371/journal.pone.0007770 (PMC2766836; doi:10.1371/journal.pone.0007770)

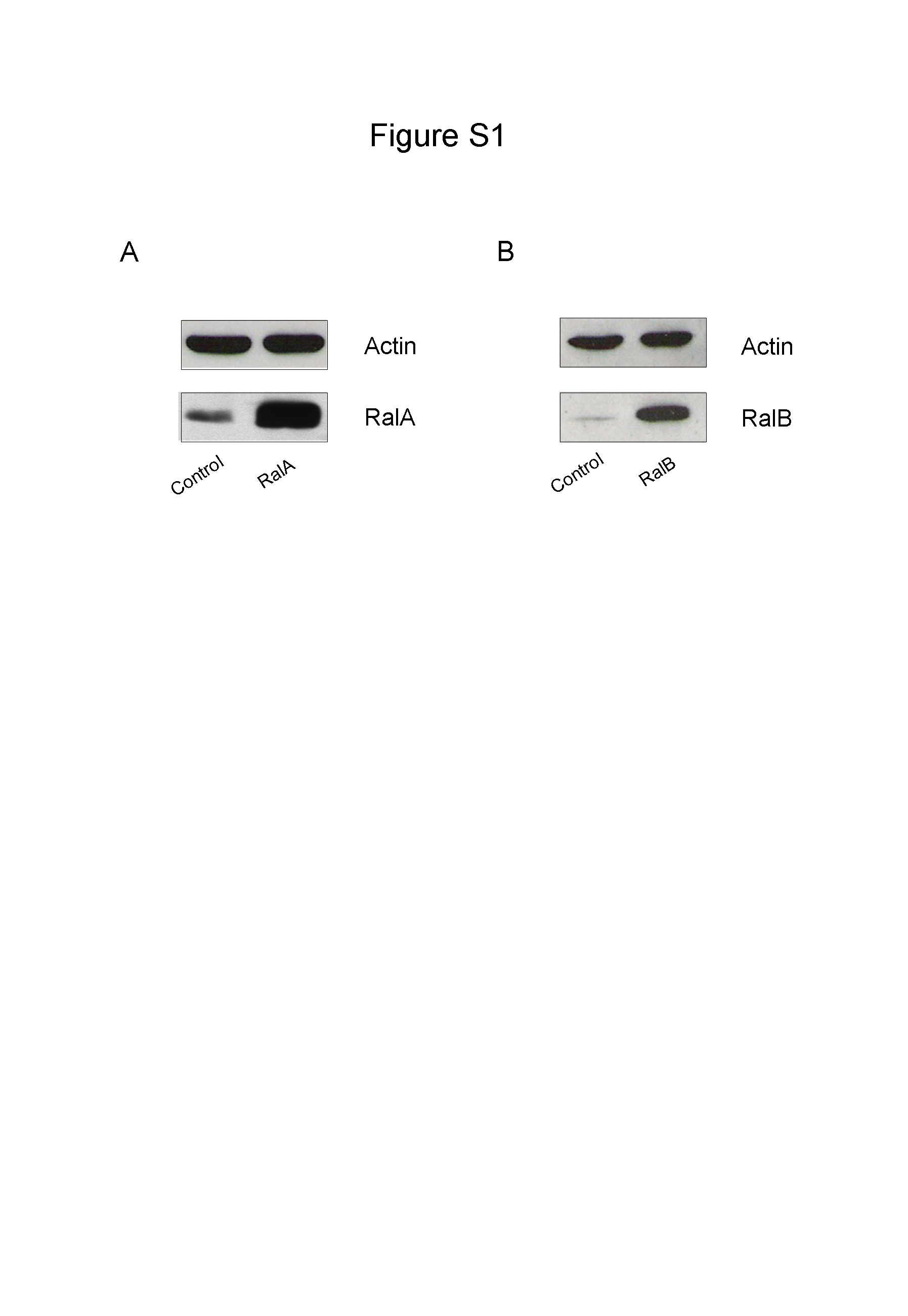

Supplement: Figure S1 — Detection of Ral GTPases in INS-1E cells overexpressing RalA or RalB. Samples obtained from INS-1E cells transfected with an empty vector (control) and from cells transfected with RalA or RalB expressing plasmids were analyzed by Western blotting using antibodies against RalA (A) or RalB (B). Equal loading between the different lanes was assessed by analyzing the same samples with an antibody against actin. (0.70 MB TIF) [file pone.0007770.s001.tif]

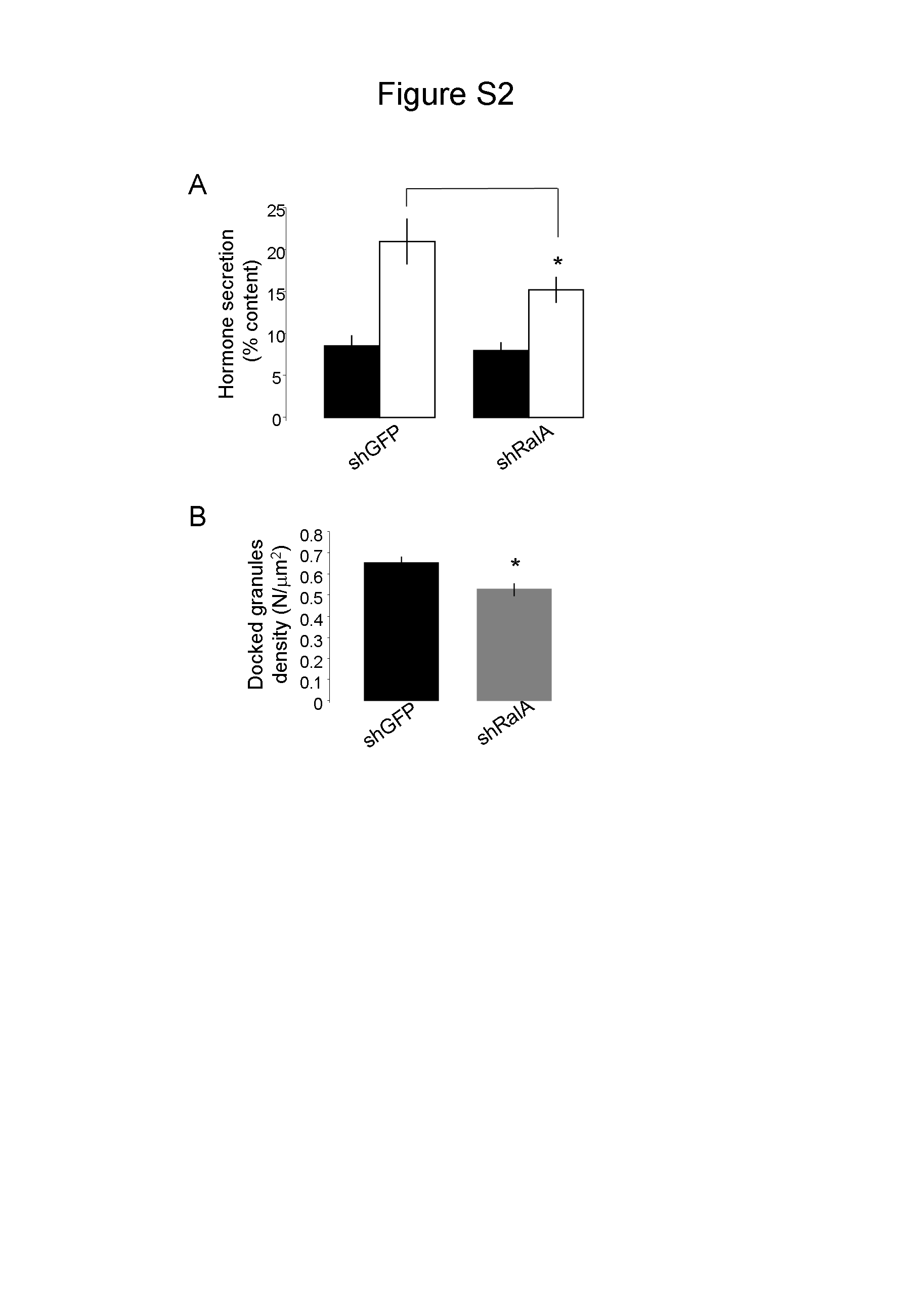

Supplement: Figure S2 — Comparison between the effect of shRalA and of a shRNA against GFP on hormone secretion and granule docking. A) INS-1E cells were co-transfected with a plasmid encoding hGH and with plasmids permitting the expression of shGFP or shRalA. Three days later the cells were incubated for 30 min at basal conditions (2 mM glucose), and then 45 min either at basal (filled bars) or stimulated conditions (20 mM glucose, 10 µM Forskolin and 100 mM IBMX, open bars). Hormone release was quantified by ELISA. The results are the means ± SEM of five independent experiments. * p<0.05 ANOVA. B) INS-1E cells were co-transfected with a plasmid encoding NPY-mRFP and either shGFP or shRalA. The number of granules docked at the plasma membrane per µm2 was observed and counted by inspecting the cells by TIRF microscopy. Silencing of RalA resulted in a significant reduction in the number of docked granules per µm2 (Bonferroni test: * p<0.05). Results are presented as means ± SEM (n = 23 cells, shGFP; n = 22 cells, shRalA). (0.46 MB TIF) [file pone.0007770.s002.tif]

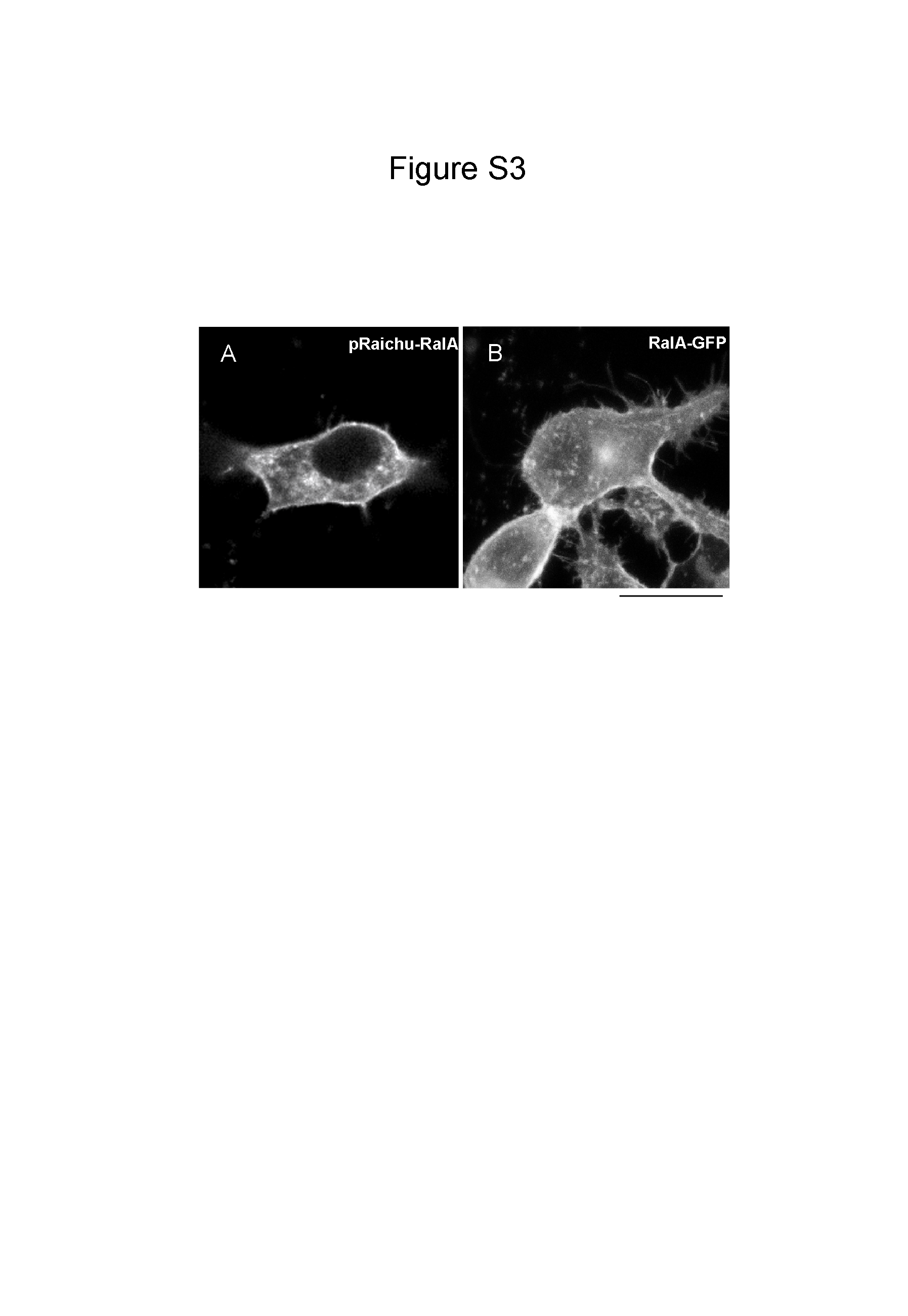

Supplement: Figure S3 — Subcellular localization of the chimeric probe pRaichu-RalA. INS-1E cells transfected with the pRaichu-RalA plasmid (A) or with GFP-tagged RalA (B) were analyzed by confocal microscopy. Notice that both fluorescent probes display a similar subcellular distribution in the cytosolic and plasma membrane compartments. Scale bar 25 µm. (1.19 MB TIF) [file pone.0007770.s003.tif]

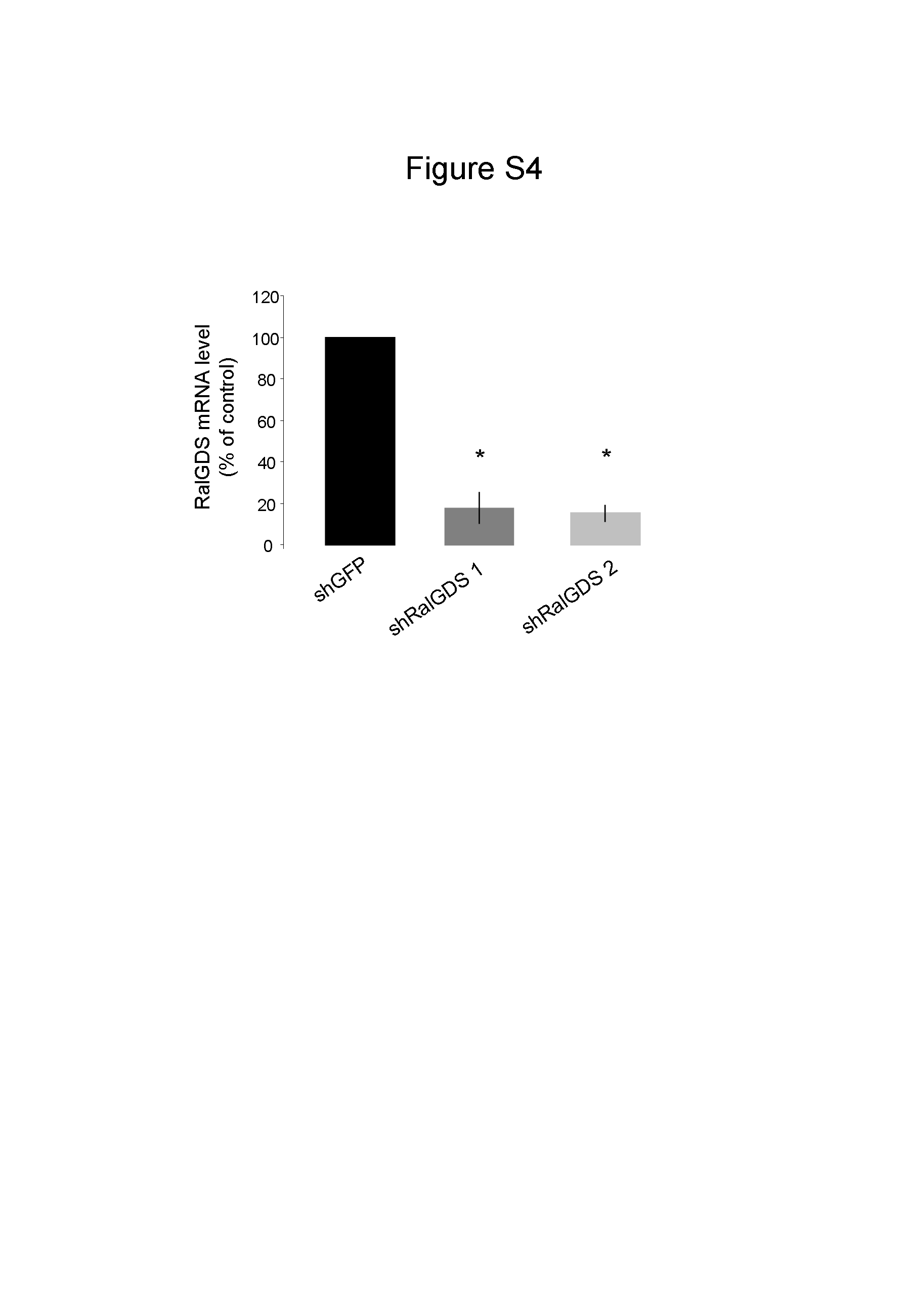

Supplement: Figure S4 — Effect of two different shRNAs on RalGDS mRNA level. To test the silencing efficiency of two shRNAs directed against RalGDS, total RNAs extracted from INS-1E cells expressing shGFP, shRalGDS1 or shRalGDS2 were analyzed by quantitative Real-Time PCR. Transfection of shRalGDS1 and shRalGDS2 reduced RalGDS mRNA levels by 83 and 85%, respectively. The results represent the means ± SD of three independent experiments. * p<0.001 ANOVA. (0.43 MB TIF) [file pone.0007770.s004.tif]

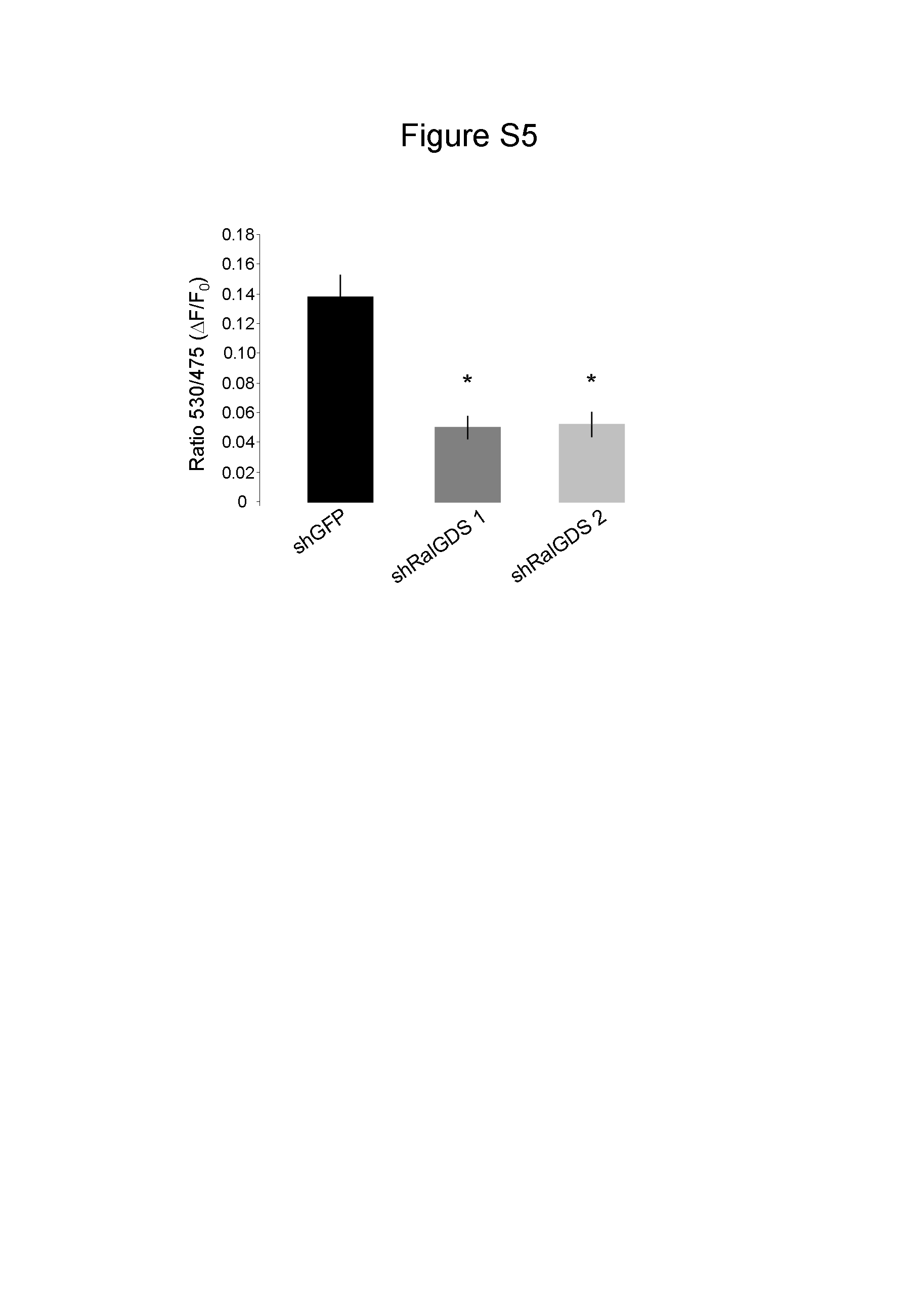

Supplement: Figure S5 — Effect of shRalGDS 1 and 2 on RalA activation. Quantification of RalA activation was performed by monitoring the variations between the fluorescence at 530 nm and 475 nm in cells transfected with shGFP, with shRalGDS1 or shRalGDS2. The results are expressed as maximum changes in the 530 nm/475 nm ratio (ΔF) divided by the value of the ratio at the beginning of the experiment (F0) ± SD (n = 6 cells shGFP; n = 4 cells shRalGDS1; n = 4 cells shRalGDS2). * p<0.05, ANOVA. (0.45 MB TIF) [file pone.0007770.s005.tif]

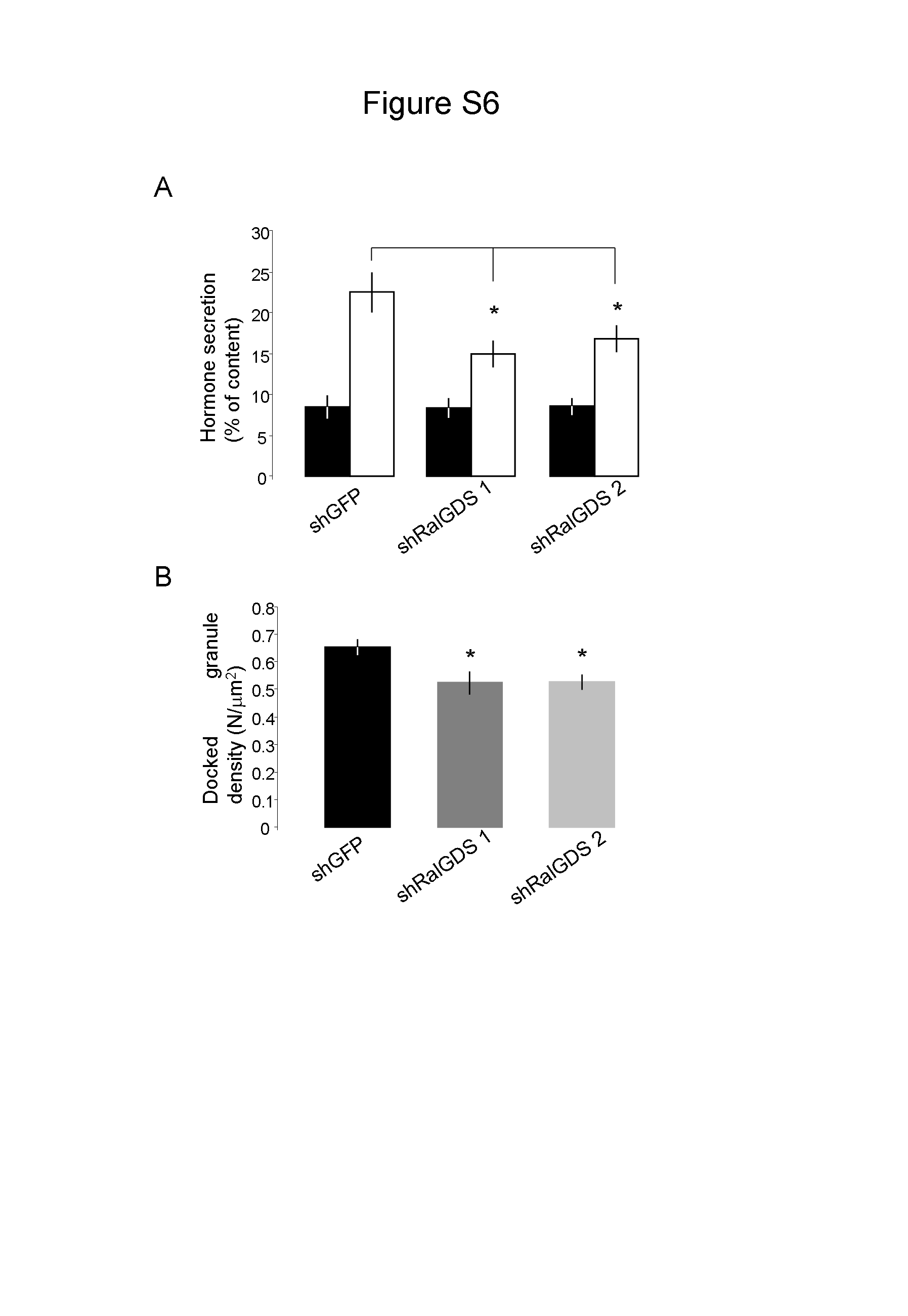

Supplement: Figure S6 — Effect of shRalGDS 1 and 2 on hormone secretion and insulin granule docking. A) INS-1E cells were co-transfected with a plasmid encoding hGH and with shGFP, shRalGDS 1 or shRalGDS 2 encoding vectors. Hormone release under basal (filled bars) and stimulatory conditions (open bars) was assessed three days later by ELISA. The results are means ± SEM of six independent experiments. * p<0.05, ANOVA. B) INS-1E cells were co-transfected with a plasmid encoding NPY-mRFP and either shGFP, shRalGDS1 or shRalGDS 2. The granules docked at the plasma membrane were visualized by TIRF microscopy. Silencing of RalGDS with shRalGDS 1 or shRalGDS 2 resulted in a significant reduction in the number of docked granules per µm2 (Bonferroni test: * p<0.05). The results are given as means ± SEM (n = 23 cells, shGFP; n = 21 cells, shRalGDS 1; n = 22 cells, shRalGDS 2). (0.50 MB TIF) [file pone.0007770.s006.tif]
